# Neuroendocrinology meets addiction: Emerging pharmacotherapies on the horizon

**DOI:** 10.1111/joim.70021

**Published:** 2025-09-23

**Authors:** Anna Loften, Mehdi Farokhnia, Leandro F. Vendruscolo, Lorenzo Leggio

**Affiliations:** ^1^ Clinical Psychoneuroendocrinology and Neuropsychopharmacology Section Translational Addiction Medicine Branch National Institute on Drug Abuse Intramural Research Program, and National Institute on Alcohol Abuse and Alcoholism Division of Intramural Clinical and Biological Research National Institutes of Health Baltimore Maryland USA; ^2^ Department of Mental Health Johns Hopkins Bloomberg School of Public Health Baltimore Maryland USA; ^3^ Stress and Addiction Neuroscience Unit Integrative Neuroscience Research Branch National Institute on Drug Abuse Intramural Research Program, and National Institute on Alcohol Abuse and Alcoholism Division of Intramural Clinical and Biological Research National Institutes of Health Baltimore Maryland USA; ^4^ Center for Alcohol and Addiction Studies Department of Behavioral and Social Sciences School of Public Health Brown University Providence Rhode Island USA; ^5^ Division of Addiction Medicine Department of Medicine School of Medicine Johns Hopkins University Baltimore Maryland USA; ^6^ Department of Neuroscience Georgetown University Medical Center Washington District of Columbia USA

**Keywords:** addiction, alcohol use disorder, amylin, FGF‐21, GLP‐1, glucocorticoid, mineralocorticoid, substance use disorder

## Abstract

Alcohol and other substance use disorders (ASUDs) are prevalent and major contributors to global morbidity and mortality. Their impact extends beyond the individual, imposing significant burdens on families, communities, healthcare systems, and society at large. Treatments include psychosocial, behavioral, and pharmacological interventions. However, available pharmacological treatments remain limited, primarily targeting alcohol, tobacco, and opioid use disorders, with a lack of approved pharmacotherapies for other substance use disorders. This gap highlights a critical need to develop novel treatment options. Emerging evidence suggests that bidirectional brain‐periphery communications play important roles in the pathophysiology and progression of ASUDs. Gut–brain hormones that are involved in the regulation of feeding and metabolism have been shown to influence reinforcing properties of food, alcohol, and other addictive substances. Additionally, stress‐related pathways, especially the hypothalamic–pituitary–adrenal axis, play a significant role in regulating behaviors that are related to ASUDs. Accordingly, feeding‐ and stress‐related neuroendocrine pathways represent novel pharmacotherapeutic targets for ASUDs. This narrative review discusses preclinical and clinical evidence for emerging pharmacotherapies that target ASUD‐related neuroendocrine systems. Special emphasis is placed on recent work with glucagon‐like peptide‐1, ghrelin, fibroblast growth factor‐21, amylin, glucocorticoids, and mineralocorticoids.

## Graphical abstract

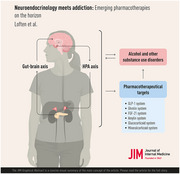

## Introduction

Alcohol and other substance use disorders (ASUDs) are chronic, relapsing conditions that contribute significantly to global morbidity and mortality. Alcohol alone is responsible for over 3 million deaths annually, accounting for approximately 5.6% of all global deaths [1]. In 2019, non‐alcohol substance‐related deaths exceeded 580,000 worldwide, with opioids being the leading cause, accounting for approximately 450,000 deaths [1]. The consequences of ASUDs expand beyond the individual, given their public health and economic consequences, affecting society as a whole.

Treatments for ASUDs include psychosocial, behavioral, and pharmacological interventions. Despite their high prevalence and burden, pharmacotherapies for ASUDs remain limited, and not all individuals respond positively to existing therapies [2]. The European Medicines Agency (EMA) and US Food and Drug Administration (FDA) have approved the following medications for the treatment of alcohol use disorder (AUD): disulfiram, acamprosate, and naltrexone. The EMA has also approved nalmefene for AUD (Table 1). Disulfiram, the earliest available of these treatments, was approved by the FDA in the early 1950s. The slow expansion of pharmacological treatment options over the past seven decades highlights the inadequate development in this area, especially relative to other chronic conditions, such as diabetes and hypertension. Additionally, some regional approvals exist. Baclofen is approved for AUD in France and is used off‐label in other countries, and sodium oxybate is approved for AUD in Italy and Austria [3]. Although the exact mechanisms remain unclear, baclofen appears to be more beneficial in patients with AUD and alcohol‐associated liver disease and is recommended by scientific liver societies for this indication [4]. Other off‐label medications with strong evidence of reducing alcohol consumption include, but are not limited to, topiramate, gabapentin, and varenicline [2, 3]. Pharmacotherapies for other substance use disorders (SUDs) are likewise scarce. Nicotine replacement therapy, bupropion, and varenicline are approved for tobacco use disorder; and buprenorphine, methadone, and naltrexone are approved for opioid use disorder (Table 1). However, no FDA‐approved medications are available for other SUDs, including stimulant and cannabis use disorders. In addition to the limited number of medications, a low proportion of individuals with ASUDs receive pharmacological treatment [1, 5]. For instance, only 1.6% of adults with AUD in the United States received pharmacological treatment in 2019 [6], representing one of the largest treatment gaps and unmet needs in healthcare. Key barriers to receiving pharmacological treatments include stigma, a lack of awareness of available options among patients and providers, heterogeneity in response to medications, limited addiction education during clinical training, and a limited number of approved medications. These challenges underscore the need for additional pharmacotherapeutic options for ASUDs.

**Table 1:** Overview of pharmacotherapies approved^a^ by the United States Food and Drug Administration (FDA) and/or the European Medicines Agency (EMA) for the treatment of alcohol, opioid, and tobacco use disorders and their primary mechanisms of action.

Indication	Pharmacotherapy	Primary mechanism(s) of action
**Alcohol use disorder**	Acamprosate	Unclear mechanism of action, but suggested mechanisms involve partial agonism of NMDA receptors as well as interaction with metabotropic glutamate, glycine, and GABA‐A receptors
	Disulfiram	Inhibition of aldehyde dehydrogenase
	Nalmefene^b^	Antagonism of μ‐ and δ‐opioid receptors and partial agonism of κ‐opioid receptors
	Naltrexone (oral and IM)	Antagonism of μ‐, κ‐, and δ‐opioid receptors
**Opioid use disorder**	Buprenorphine	Partial agonism of μ‐opioid and antagonism of κ‐ and δ‐opioid receptors
	Naltrexone (IM)	Antagonism of μ‐, κ‐, and δ‐opioid receptors
	Methadone	Agonism of μ‐opioid receptors; additional mechanisms include antagonism of NMDA receptors and inhibition of serotonin and norepinephrine reuptake
**Tobacco use disorder**	Bupropion	Inhibition of norepinephrine and dopamine reuptake
	Nicotine replacement therapy	Agonism of nicotinic receptors
	Varenicline	Partial agonism of α4β2 nicotinic acetylcholine receptors

Abbreviation: IM, intramuscular.

^a^Pharmacotherapies to treat withdrawal or overdose are not included in this table.

^b^ Approved by EMA only.

Chronic substance misuse leads to neuroadaptive changes in the brain, which affect a variety of neurocircuitries and neurotransmitter systems in a complex, interconnected, and yet not fully understood manner [7]. The consequences of substance use extend beyond the brain, affecting several organs and triggering adaptive responses that lead to various medical complications. Thus, ASUDs can be viewed as systemic diseases. Growing evidence suggests that brain‐periphery communications, including neuroendocrine systems, play important roles in the pathophysiology and progression of ASUDs.

Alcohol is caloric and has palatable properties. Alcohol intake shares common neurobiological mechanisms with food intake behaviors, including, but not limited to, those related to reward processing [8]. Gut–brain hormones that regulate feeding and metabolism have been shown to influence rewarding properties of food, alcohol, and other addictive substances. Similarly, stress‐related pathways, particularly the hypothalamic–pituitary–adrenal (HPA) axis, play a significant role in regulating food intake and addictive behaviors [9]. Accordingly, feeding‐ and stress‐related endocrine pathways may serve as novel pharmacotherapeutic targets for ASUDs.

The present review discusses preclinical and clinical data on putative pharmacotherapies that target neuroendocrine systems involved in ASUDs, with a focus on new promising targets, such as glucagon‐like peptide‐1 (GLP‐1), ghrelin, fibroblast growth factor‐21 (FGF‐21), amylin, glucocorticoids, and mineralocorticoids (Table 2). Evidence of the potential utility of targeting these pathways is generally more extensive for AUD than for other SUDs. Given the scope of this review, detailed descriptions of methods and statistical analyses are not provided but can be found in other review articles (e.g., [10, 11, 12, 13]). Studies were selected through keyword searches (Table 3) in PubMed and Web of Science. A snowball search was also employed when appropriate.

**Table 2:** Overview of the neuroendocrine systems discussed in this review and availability of data for addictive substances.

Neuroendocrine system	Addictive substance	Preclinical data available	Clinical data available
Glucagon‐like peptide‐1	Alcohol	**✓**	**✓**
	Psychostimulants	**✓**	**✓**
	Opioids	**✓**	**✓**
	Nicotine	**✓**	**✓**
	Cannabis		**✓**
Ghrelin	Alcohol	**✓**	**✓**
	Psychostimulants	**✓**	**✓**
	Opioids	**✓**	**✓**
	Nicotine	**✓**	**✓**
	Cannabis	**✓**	**✓**
Fibroblast growth factor 21	Alcohol	**✓**	**✓**
	Opioids	**✓**	
	Cannabis	**✓**	
Amylin	Alcohol	**✓**	
	Stimulant	**✓**	**✓**
	Nicotine	**✓**	
Glucocorticoid	Alcohol	**✓**	**✓**
	Psychostimulants	**✓**	
	Opioids	**✓**	**✓**
	Nicotine	**✓**	
Mineralocorticoid	Alcohol	**✓**	**✓**
	Psychostimulants	**✓**	
	Opioids	**✓**	

**Table 3:** Keywords used for literature search in PubMed and web of science.

Keywords alcohol and other substances	Addiction, alcohol, alcohol addiction, alcohol use disorder, amphetamine, cannabis, cannabis use disorder, cocaine, ethanol, heroin, morphine, nicotine, opioids, opioid use disorder, substance use disorder
Gut peptides	Amylin, dipeptidyl peptidase‐4, dulaglutide, exenatide, fibroblast growth factor 21, ghrelin, glucose‐dependent insulinotropic polypeptide, glucagon‐like peptide 1, liver‐expressed antimicrobial peptide‐2, liraglutide, semaglutide, tirzepatide
Stress system	Aldosterone, corticosterone, cortisol, glucocorticoid receptors, hypothalamic–pituitary–adrenal axis, mifepristone, mineralocorticoid receptors, spironolactone, stress

## Glucagon‐like peptide‐1 system

**Fig. 1:**
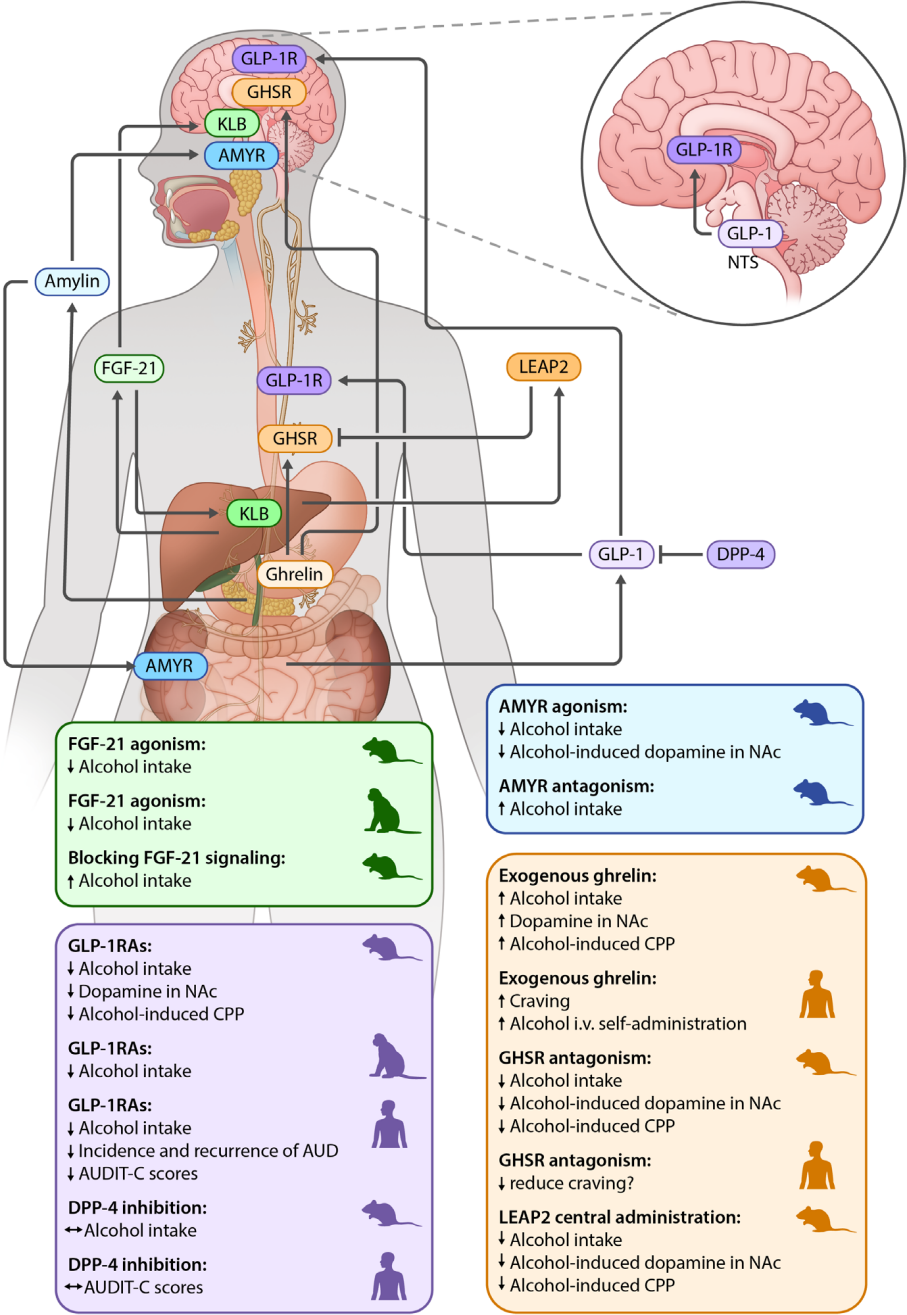
Simplified overview of the gut–brain axis and their role in alcohol‐related outcomes. Glucagon‐like peptide‐1 (GLP‐1) is mainly produced in the intestines but also centrally in the nucleus tractus solitarius (NTS) and acts via GLP‐1 receptors (GLP‐1Rs) both peripherally and centrally. Circulating GLP‐1 is degraded by dipeptidyl peptidase‐4 (DPP‐4). Ghrelin is mainly produced by the stomach, where it is released into the circulation. Ghrelin exerts its effect by acting on growth hormone secretagogue receptors (GHSRs) that are expressed in the periphery, including on the vagus nerve, and centrally. Liver‐expressed antimicrobial peptide‐2 (LEAP2) is produced by the liver and blocks GHSRs by acting as an inverse agonist. Fibroblast growth factor‐21 (FGF‐21) is mainly produced by the liver and acts on receptors both in the periphery and centrally. Amylin is primarily produced by pancreatic β cells and acts on amylin receptors (AMYRs) peripherally and centrally. AUD, alcohol use disorder; AUDIT‐C, Alcohol Use Disorder Identification Test—Consumption; CPP, conditioned place preference; GLP‐1RAs, GLP‐1R agonists; i.v., intravenously; KLB, β‐klotho; NAc, nucleus accumbens.

GLP‐1 is an incretin peptide with anorexigenic properties. It is produced primarily by L‐cells in the small intestines [14]. Circulating GLP‐1 is quickly degraded by dipeptidyl peptidase‐4 (DPP‐4), resulting in a short half‐life of approximately 2 min [15]. GLP‐1 regulates glucose homeostasis by enhancing insulin secretion and suppressing glucagon release [16]. Based on these physiological effects, pharmacotherapies that activate GLP‐1 receptors (GLP‐1‐Rs) or inhibit DPP‐4 have been developed for the treatment of Type 2 diabetes. More recently, some GLP‐R agonists (GLP‐1RAs) have also been approved for the treatment of obesity, based on their effectiveness in reducing appetite and food intake and promoting weight loss, as well as for obstructive sleep apnea and metabolic dysfunction‐associated steatohepatitis. Compared with first‐generation GLP‐1RAs such as exenatide, newer ones such as semaglutide are longer acting and have higher receptor affinity [17]. In addition to the periphery, GLP‐1 is also expressed in the brain, mainly in the nucleus tractus solitarius (NTS) and to a lesser extent in the medullary reticular formation [18]. Projections from the NTS target brain regions associated with reward and reinforcement, including the nucleus accumbens (NAc), ventral tegmental area (VTA), and the laterodorsal tegmental area (LDTg). GLP‐1 mediates its effects via GLP‐1Rs that are expressed both peripherally and centrally [19] (Figure 1).

### GLP‐1 and alcohol use

Several GLP‐1RAs have been evaluated for their potential to influence alcohol intake using various alcohol consumption paradigms in animal models. In the two‐bottle choice paradigm, lower alcohol consumption has been observed in rats following treatment with exenatide [20, 21], liraglutide [22, 23], dulaglutide [24], and semaglutide [23, 25]. In operant settings, mice that were treated with exenatide exhibited a reduction of intravenous alcohol self‐administration [26], and rats that were treated with liraglutide and semaglutide exhibited a reduction of oral alcohol self‐administration [22, 27]. In the drinking‐in‐the‐dark paradigm, which models binge‐like drinking, a decrease in alcohol intake was observed in mice following treatment with semaglutide [27]. Relapse‐like drinking is often modeled using the alcohol deprivation effect (ADE) paradigm. A blunted ADE was observed in mice that were treated with exenatide [28] and in rats that were treated with liraglutide [22] or semaglutide [25]. In addition to rodent models, in male alcohol‐preferring velvet monkeys, alcohol consumption decreased following treatment with exenatide, liraglutide, or semaglutide [29, 30]. The effect on alcohol consumption is believed to involve central mechanisms. Administration of exenatide in the NTS [31], NAc [32, 33], VTA [33], LDTg [32], dorsolateral septum [34], dorsal hippocampus, and lateral hypothalamus [33] decreased alcohol intake in rodents. The reduction of alcohol intake is thought to be at least partially linked to an effect in lowering alcohol‐related rewarding effects. Specifically, alcohol‐induced dopamine release is attenuated by exenatide, liraglutide, and semaglutide, which demonstrated both directly via in vivo microdialysis [21, 25, 31] and indirectly via a reduction of alcohol‐induced locomotor stimulation [21, 25]. Additionally, GLP‐1RAs reduce alcohol‐induced conditioned place preference (CPP) in rodents [21, 22], further suggesting a reduction of alcohol‐related rewarding effects (Table 4).

**Table 4:** Summary of key alcohol‐related outcomes from reviewed preclinical studies.

Pharmacotherapy	Species	Experimental procedure/Behavioral paradigm	Outcome	References
**GLP‐1 pathway**				
Exenatide	Mouse	Operant intravenous alcohol self‐administration	↓ Self‐administration of intravenous alcohol following exenatide treatment compared to vehicle	[26]
		Operant self‐administration using a PR test, treatment effect was assessed after 9 months of voluntary alcohol exposure	↓ Alcohol self‐administrated (lever presses for alcohol) and motivation for alcohol (PR breaking point) following exenatide treatment compared to vehicle	[21]
		Alcohol deprivation effect model following a period of forced abstinence	↓ Relapse‐like drinking following exenatide treatment compared to vehicle	[28]
		Alcohol‐induced CPP	↓ Alcohol‐induced CPP after both acute and chronic exenatide treatment	[21]
		In vivo microdialysis to measure dopamine in the NAc	↓ Alcohol‐induced dopamine increase in the NAc following exenatide treatment compared to vehicle	[21]
	Rat	Two‐bottle choice intermittent access to 20% alcohol for three 24‐h sessions per week for 4 weeks or 8 months before exenatide	↓ Alcohol intake and preference following exenatide treatment compared to vehicle, both after short‐ and long‐term exposure to alcohol prior to start of treatment	[20, 21]
	NHP	Two‐bottle choice paradigm with alcohol access 4 h/day with once weekly exenatide for 5 weeks	↓ Alcohol intake following exenatide treatment compared to placebo	[30]
Liraglutide	Rat	Two‐bottle choice intermittent access to 10%–20% alcohol for three 24‐h sessions per week	↓ Alcohol intake following liraglutide treatment compared to vehicle in two independent studies	[22, 23]
		Operant self‐administration in selectively bred alcohol‐preferring sP rats	↓ Alcohol intake following liraglutide treatment compared to vehicle	[22]
		Alcohol deprivation effect model following a period of forced abstinence	↓ Relapse‐like drinking following acute liraglutide treatment compared to vehicle	[22]
		Alcohol‐induced CPP	↓ Alcohol‐induced CPP after treatment with liraglutide compared to vehicle	[22]
		In vivo microdialysis to measure dopamine in the NAc	↓ Alcohol‐induced dopamine increase in the NAc following liraglutide treatment compared to vehicle	[22]
	NHP	Two‐bottle choice paradigm with alcohol access 4 h/day with daily liraglutide for 2 weeks	↓ Alcohol intake following liraglutide treatment compared to placebo	[30]
Dulaglutide	Rat	Two‐bottle choice intermittent access to 20% alcohol for three 24‐h sessions per week with dulaglutide once weekly for 5 or 9 weeks	↓ Alcohol intake and preference following dulaglutide treatment compared to vehicle, both after 5 and 9 weeks of treatment	[24]
Semaglutide	Mouse	Drinking‐in‐the‐dark paradigm using sweetened and unsweetened alcohol solutions	↓ Intake of both sweetened and unsweetened alcohol solutions following semaglutide treatment compared to vehicle	[27]
		In vivo microdialysis to measure dopamine in the NAc	↓ Alcohol‐induced dopamine increase in the NAc following semaglutide treatment compared to vehicle	[25]
	Rat	Two‐bottle choice intermittent access to 10%–20% alcohol for three 24‐h sessions per week	↓ Alcohol intake following semaglutide treatment in two independent studies	[23, 25]
		Operant self‐administration using an FR1 schedule with sweetened and unsweetened alcohol solutions	↓ Intake of both sweetened and unsweetened alcohol solutions following semaglutide treatment compared to vehicle	[27]
		Alcohol deprivation effect model following a period of forced abstinence	↓ Relapse‐like drinking following semaglutide treatment compared to vehicle	[25]
	NHP	Two‐bottle choice paradigm with alcohol access 4 h/day with twice‐weekly semaglutide	↓ Alcohol intake following semaglutide treatment compared to placebo	[29]
**Ghrelin pathway**				
Ghrelin (agonist)	Mouse	Two‐bottle choice paradigm with access to 10%–15% alcohol 90 min/day, with ghrelin administered into the VTA, LDTg or ICV	↑ Alcohol intake following ghrelin administration to all locations tested compared to vehicle	[75, 76]
		In vivo microdialysis to measure dopamine in the NAc following ICV administration of ghrelin	↑ Dopamine in the NAc following ICV ghrelin administration compared to vehicle	[89]
JMV2959 (antagonist)	Mouse	Two‐bottle choice paradigm with continuous access to 10% alcohol	↓ Alcohol intake following JVM2959 treatment compared to vehicle	[80]
		Two‐bottle choice with access to 15% alcohol solution for 2 h/day	↓ Alcohol intake following JVM2959 treatment compared to vehicle	[79]
		Drinking‐in‐the‐dark paradigm using sweetened and unsweetened alcohol solutions	↓ Intake of both sweetened and unsweetened alcohol solutions following JVM2959 treatment compared to vehicle	[82]
		Alcohol deprivation effect model following a period of forced abstinence	↓ Relapse‐like drinking following JMV2959 treatment compared to vehicle	[80]
		In vivo microdialysis to measure dopamine in the NAc	↓ Alcohol‐induced dopamine increase in the NAc following JVM2959 treatment compared to vehicle	[75]
		Alcohol‐induced CPP	↓ Alcohol‐induced CPP following JVM2959 treatment compared to vehicle	[75]
	Rat	Operant self‐administration	↓ Alcohol self‐administration (lever presses for alcohol) following JVM2959 treatment compared to vehicle	[77, 79]
		Two‐bottle choice intermittent access to 20% alcohol for three 24‐h sessions per week	↓ Alcohol intake and preference following JVM2959 treatment compared to vehicle, with bigger treatment effect following 5 months of alcohol exposure compared to 2 months	[77, 78]
		Alcohol deprivation effect model following a period of forced abstinence	↓ Relapse‐like drinking following JVM2959 treatment in rats exposed to alcohol for 8 months compared to vehicle	[78]
	Prairie voles	Two‐bottle choice limited access to a 20% alcohol solution for 2 h/day	↓ Alcohol intake and preference following JVM2959 treatment compared to vehicle	[81]
PF‐5190457 (inverse agonist)	Mouse	Drinking‐in‐the‐dark paradigm using sweetened and unsweetened alcohol solutions	↓ Intake of both sweetened and unsweetened alcohol solutions following PF‐5190457 treatment compared to vehicle	[82]
		Two‐bottle choice with continues access to 15% alcohol solution	↓ Alcohol intake and preference following PF‐5190457 treatment compared to vehicle	[83]
LEAP2 (inverse agonist)	Mouse	Drinking‐in‐the‐dark paradigm using sweetened and unsweetened alcohol solutions	↔ Intake of both sweetened and unsweetened alcohol solutions following systemic LEAP2 treatment compared to vehicle ↓ Intake of sweetened alcohol solution after ICV administration of LEAP2 compared to vehicle	[82]
	Rat	Two‐bottle choice intermittent access to 20% alcohol for three 24‐h sessions per week	↓ Alcohol intake following ICV administration of LEAP2 compared to vehicle	[91]
		In vivo microdialysis to measure dopamine in the NAc with ICV administration of LEAP2 followed by systemic administration of alcohol	↓ Alcohol‐induced dopamine increase in the NAc following ICV LEAP2 administration compared to vehicle	[91]
**FGF‐21 pathway**				
FGF‐21	Mouse	Two‐bottle choice continuous access to alcohol solutions ranging between 2% and 16% across two studies	↓ Alcohol intake following FGF‐21 treatment for all alcohol concentrations except the lowest one of 2%, compared to vehicle	[128, 136]
PF‐05231023	Mouse	Two‐bottle choice with intermittent access to 20% alcohol for three 24‐h sessions per week	↓ Alcohol intake following PF‐05231023 treatment compared to vehicle	[128]
	NHP	Two‐bottle choice with limited access to 10% alcohol for 4 h/day	↓ Alcohol intake following PF‐05231023 treatment compared to placebo	[128]
**Amylin pathway**				
sCT	Mouse	In vivo microdialysis to measure dopamine in the NAc	↓ Alcohol‐induced dopamine increase in the NAc following sCT treatment compared to vehicle	[147]
		Alcohol‐induced CPP	↓ Alcohol‐induced CPP following sCT treatment compared to vehicle	[147]
	Rat	Two‐bottle choice with intermittent access to 20% alcohol for three 24‐h sessions per week	↓ Alcohol intake and preference following sCT treatment compared to vehicle	[147, 148]
		Alcohol deprivation effect model following a period of forced abstinence	↓ Relapse‐like drinking following sCT treatment compared to vehicle	[148]
AMY1213	Rat	Two‐bottle choice intermittent access to 20% alcohol for three 24‐h sessions per week	↓ Alcohol intake following AMY1213 treatment compared to vehicle	[149]
**HPA axis**				
Spironolactone	Mouse	Drinking‐in‐the‐dark paradigm using sweetened and unsweetened alcohol solutions	↓ Intake of both sweetened and unsweetened alcohol solutions following spironolactone treatment compared to vehicle	[184]
	Rat	Operant self‐administration with FR1 and FR2 schedules	↓ Alcohol self‐administration (lever presses for alcohol) following spironolactone treatment compared to vehicle	[183, 184]
Mifepristone	Mouse	Drinking‐in‐the‐dark paradigm using high‐drinking‐in‐the‐dark mice selectively bred for high alcohol intake in the specific paradigm	↓ Alcohol intake following mifepristone treatment compared to vehicle	[162]
	Rat	Two‐bottle choice limited access to 10% alcohol solution for 1 h/day	↓ Alcohol intake following mifepristone treatment compared to vehicle	[159]
		Operant self‐administration with FR1 schedule	↓ Alcohol self‐administration (lever presses for alcohol) following mifepristone treatment compared to vehicle	[160, 163, 166]
	NHP	Two‐bottle choice paradigm with access to 4% alcohol solution for 22 h per day for 6 months	↓ Alcohol intake following mifepristone treatment compared to baseline	[168]
CORT113176	Mouse	Drinking‐in‐the‐dark paradigm using high‐drinking‐in‐the‐dark mice selectively bred for high alcohol intake	↓ Alcohol intake following CORT113176 treatment compared to vehicle	[162]
	Rat	Operant self‐administration with FR1 schedule	↓ Alcohol self‐administration (lever presses for alcohol) following CORT113176 treatment compared to vehicle	[160, 163]

*Note*: Unless otherwise stated, findings refer to outbred animal models and involve systemic, daily drug administration.

Abbreviations: AUD, alcohol use disorder; AUDIT‐C, Alcohol Use Disorder Identification Test—Consumption; BMI, body mass index; CPP, conditioned place preference; DPP‐4, dipeptidyl peptidase‐4; FR, Fixed ratio; FGF‐21, fibroblast growth factor 21; GLP‐1, glucagon‐like peptide 1; HPA, hypothalamic–pituitary–adrenal axis; ICV, intracerebroventricular; LDTg, laterodorsal tegmental area; LEAP2, liver‐expressed antimicrobial peptide 2; NAc, nucleus accumbens; NHP, nonhuman primate; PR, progressive ratio; RCT, randomized clinical trial; sCT, salmon calcitonin; sP, Sardinian alcohol‐preferring rats; VTA, ventral tegmental area.

In humans, genetic variations that affect GLP‐1R function have been associated with the risk, severity, and brain correlates of AUD [35, 36]. A human postmortem study showed higher GLP‐1R mRNA expression in the hippocampus and prefrontal cortex in male individuals with AUD compared with controls [37]. Pharmacoepidemiological studies that analyzed electronic health records consistently show that prescriptions for GLP‐1RAs for diabetes and/or obesity are associated with a lower incidence and recurrence of AUD, fewer alcohol‐related events, and lower AUD Identification Test—Consumption (AUDIT‐C) scores compared with propensity‐scored controls [38, 39, 40, 41, 42, 43]. The first randomized controlled trial (RCT) that studied the effect of a GLP‐1RA in individuals with AUD examined extended‐release exenatide. No effect on alcohol consumption was found in the full sample, but a secondary analysis suggested that exenatide reduced alcohol consumption in a subset of individuals with AUD and obesity (body mass index [BMI] > 30 kg/m^2^) [44]. A recent RCT found that low‐dose semaglutide reduced the amount of alcohol that was consumed under a laboratory self‐administration task, the number of drinks consumed on drinking days, and weekly craving ratings during the study [45] (Table 5).

**Table 5:** Summary of key alcohol‐related outcomes from reviewed human studies.

Pharmacotherapy	Study design	Outcome	References
**GLP‐1 pathway**			
Exenatide	RCT, treatment‐seeking individuals with AUD (*n* = 127) receiving exenatide once weekly for 26 weeks, primary outcome: heavy drinking days as per TLFB	↓ Heavy drinking days in a subset of study participants having a BMI ≥30 following exenatide treatment compared to placebo, with no significant difference in the full sample	[44]
Semaglutide	RCT, nontreatment‐seeking individuals with AUD (*n* = 48) receiving semaglutide (0.25 mg/week, 0.5 mg/week for 4 weeks, and 1 mg for 1 week) or placebo weekly for 9 weeks, primary outcome: laboratory alcohol self‐administration, secondary outcomes included alcohol intake assessed by TLFB	↓ Alcohol intake during posttreatment laboratory self‐administration following semaglutide treatment compared to placebo. Semaglutide significantly reduced the number of drinks consumed per drinking day and weekly alcohol craving ratings	[45]
	Retrospective cohort study using electronic health record data from the United States Department of Veterans Affairs, primary outcome: change in AUDIT‐C scores	↓ Alcohol intake, as indicated by decreased AUDIT‐C scores, in individuals prescribed semaglutide compared to propensity‐score‐matched controls	[38]
	Retrospective cohort study using electronic health record data from the TriNetX Analytics Network	↓ Incidence and recurrence of AUD in individuals prescribed semaglutide compared to propensity‐score‐matched controls	[39]
	A case series of six patients with AUD who initiated semaglutide therapy for obesity, outcome measure was change in AUDIT‐C scores	↓ Alcohol intake, indicated by decreased AUDIT‐C scores, in all participants	[191]
**Ghrelin pathway**			
Ghrelin (agonist)	RCT, nontreatment‐seeking individuals with AUD (*n* = 45) receiving intravenous ghrelin (1 µg/kg or 3 µg/kg), followed by a cue‐reactivity test, primary outcome: alcohol craving assessed with A‐VAS	↑ Cue‐induced alcohol craving, indicated by decreased A‐VAS scores, following ghrelin administration compared to placebo, while no effect was seen on cue‐induced craving for juice or food	[102]
	RCT, crossover laboratory study with nontreatment‐seeking heavy‐drinking individuals with AUD receiving a bolus of intravenous ghrelin (3 µg/kg) followed by continuous ghrelin (16.9 ng/kg/min), primary outcome: alcohol self‐administration in an intravenous self‐administration paradigm	↑ Number of alcohol infusions self‐administered and shorter duration until initiation of alcohol self‐administration, following ghrelin infusion compared to placebo	[103]
PF‐5190457 (inverse agonist)	Single‐blind, placebo‐controlled, within‐subject clinical trial in heavy‐drinking individuals with AUD (*n* = 12), receiving PF‐5190457 (100 mg/day and 200 mg/day), primary outcome: safety and alcohol/drug interaction; secondary outcome measures included craving measured during a cue‐reactivity test and AUQ scores	↓ Cue‐induced alcohol craving, indicated by decreased AUQ‐scores, following PF‐5190457 treatment compared to placebo, with no detected alcohol/drug interaction and only mild to moderate drug‐related adverse events	[104]
	RCT, within‐subject, treatment‐seeking individuals with AUD (*n* = 42) receiving PF‐5190457 (200 mg/day), primary outcome: cue‐elicited craving assessed with a cue‐reactivity test and AUQ scores	↔ Cue‐induced alcohol craving, indicated by no significant change in AUQ‐scores, following treatment with PF‐5190457 compared to placebo	[105]
**HPA axis**			
Spironolactone	Retrospective cohort study using electronic health record data from the United States Department of Veterans Affairs	↓ Alcohol intake, indicated by decreased AUDIT‐C scores, in individuals prescribed spironolactone compared to propensity‐score‐matched controls	[184]
	Retrospective cohort study using patient electronic health record data from Kaiser Permanente Northern California	↓ Alcohol intake, indicated by decreased alcohol drinks per week, following initiation of spironolactone treatment compared to propensity‐score‐matched controls	[188]
Mifepristone	RCT, nontreatment‐seeking individuals with AUD (*n* = 56) received oral mifepristone (600 mg daily) or placebo for 1 week, primary outcome: alcohol craving measured by A‐VAS, during a cue‐reactivity test; secondary outcomes included alcohol intake assessed by TLFB	↓ Cue‐induced alcohol craving, indicated by decreased A‐VAS scores, and reduced alcohol intake following mifepristone treatment compared to placebo	[163]
	RCT, within‐subject, nontreatment‐seeking individuals with AUD (*n* = 32) received oral mifepristone (600 mg daily) for 1 week, secondary outcomes included craving measured using ACQ and alcohol intake in a bar laboratory self‐administration session	↓ Cue‐induced craving, indicated by decreased ACQ‐scores, following mifepristone treatment compared to placebo	[172]

Abbreviations: ACQ, alcohol craving questionnaire; AUD, alcohol use disorder; AUDIT‐C, Alcohol Use Disorder Identification Test—Consumption; AUQ, alcohol urge questionnaire; A‐VAS, alcohol visual analogue scale; BMI, body mass index; GLP‐1, glucagon‐like peptide‐1; HPA, hypothalamic–pituitary–adrenal axis; RCT, randomized clinical trial; TLFB, timeline following back.

In addition to GLP‐1RAs, some studies have examined the effects of increasing endogenous GLP‐1 levels by inhibiting DPP‐4. The concomitant administration of alcohol and a DPP‐4 inhibitor delayed the development of tolerance to anxiolytic‐like effects of alcohol and delayed the appearance of withdrawal‐induced anxiety‐like behavior in rats [46]. However, no changes in alcohol consumption were observed following treatment with DPP‐4 inhibitors in rodents [23, 38]. In transgenic mice with a deficiency in the DPP‐4 gene, no changes in alcohol preference were observed compared with controls [47] (Table 4). Pharmacoepidemiological human data further indicate that prescriptions of DPP‐4 inhibitors are not associated with changes in alcohol intake [38, 40] (Table 5). Hence, unlike GLP‐1R agonism, increasing endogenous GLP‐1 by inhibiting its degradation has so far failed to show an effect on alcohol consumption.

Collectively, the existing data robustly support the effect of GLP‐1RAs in reducing alcohol consumption across various drinking models and species. The finding that GLP‐1RAs are effective in various consumption paradigms strengthens their clinical potential. Alcohol use disorder is a highly heterogeneous condition, and patients present with different phenotypes and drinking patterns. Initial preclinical studies investigated short‐acting GLP‐1RAs, and subsequent research investigated the effect of longer‐acting and more potent compounds. An effect has been observed across all GLP‐1RAs that have been tested, but the effect on alcohol‐related outcomes appears to be more robust with newer long‐acting GLP‐1RAs, such as dulaglutide and semaglutide. Long‐acting GLP‐1RAs have, in addition to superior efficacy, the advantage of less frequent dosing that may improve adherence. The most studied of these compounds is semaglutide, and several ongoing RCTs are evaluating the effect of semaglutide on alcohol‐related outcomes (Figures 1, 3).

Semaglutide is clinically used to treat obesity, leading to a significant decrease in body weight. Thus, from a safety standpoint, it may not be suitable for individuals with malnutrition and/or low body weight. However, initial findings suggest a reduction of alcohol intake by a dose of semaglutide lower than what is often used for weight loss [45]. This suggests that dose adjustments may be required to tailor the treatment to individual needs. The effect of exenatide was only shown in individuals with AUD and obesity, whereas greater intake was seen in individuals with a lower BMI [44]. The same was not shown for semaglutide [45]. Further research is needed to evaluate the impact of GLP‐1RAs on AUD in individuals with various metabolic and body composition states, among other potential predictors of response. A higher risk of medication‐induced pancreatitis has been reported in patients who were treated with GLP‐1RAs [48]. However, a recent meta‐analysis challenges this [49]. Excessive alcohol consumption itself is a well‐known causal risk factor for both acute and chronic pancreatitis, and this risk needs to be monitored in AUD populations. The reduction of alcohol intake that is observed with GLP‐1RAs may provide a net benefit that outweighs the potential risks.

### GLP‐1 and other substance use disorders

GLP‐1 therapies have also been studied for other SUDs, although to a lesser extent. The administration of GLP‐1RAs, systemically or centrally in reward‐related brain areas, decreased the self‐administration of cocaine [50, 51, 52, 53, 54], heroin [55], fentanyl [56], oxycodone [57], and nicotine [58] in various consumption paradigms in rodents but had no effect on intravenous remifentanil self‐administration [59]. Moreover, GLP‐1RAs reduced cue‐, drug‐, and/or stress‐induced reinstatement of cocaine, heroin, fentanyl, and nicotine self‐administration [50, 51, 52, 55, 58, 60]. Consistent with the alcohol data, GLP‐1RAs attenuate dopamine release in the NAc induced by amphetamine [61], cocaine [53, 61], and nicotine [62]. Moreover, GLP‐1RAs blocked CPP induced by amphetamine [61] and cocaine [61] but not by morphine [59] in rodents (Table 6).

**Table 6:** Summary of key drug‐related outcomes from reviewed preclinical studies.

Pharmacotherapy	Species	Experimental procedure/Behavioral paradigm	Outcome	References
**GLP‐1 pathway**				
Exenatide	Mouse	Operant intravenous self‐administration of drugs	↓ Cocaine intake following exenatide treatment compared vehicle in an FR1 schedule	[53]
			↔ Remifentanil intake following exenatide treatment compared vehicle in an FR1, FR3, and FR5 schedule	[59]
		Drug‐induced CPP	↔ Morphine‐induced CPP following exenatide treatment compared to vehicle	[59]
			↓ Cocaine‐induced CPP following exenatide treatment compared to vehicle	[61]
			↓ Amphetamine‐induced CPP following exenatide treatment compared to vehicle	[61]
			↓ Nicotine‐induced CPP following exenatide treatment compared to vehicle	[62]
	Rat	Operant intravenous self‐administration of drugs	↓ Cocaine reinstatement following exenatide treatment compared to vehicle across independent studies	[50, 51, 52]
			↓ Fentanyl intake and drug‐induced reinstatement following exenatide treatment compared to vehicle	[56]
			↓ Oxycodone intake and drug‐induced reinstatement following exenatide treatment compared to vehicle	[57]
		In vivo microdialysis to measure dopamine in the NAc	↓ Cocaine‐induced dopamine increase in the NAc following exenatide treatment compared to vehicle	[53, 61]
			↓ Amphetamine‐induced dopamine increase in the NAc following exenatide treatment compared to vehicle	[61]
			↓ Nicotine‐induced dopamine increase in the NAc following exenatide treatment compared to vehicle	[62]
Liraglutide	Rat	Operant intravenous self‐administration of drugs	↓ Heroin intake and drug‐induced reinstatement following liraglutide treatment compared to vehicle	[55]
			↓ Fentanyl‐induced reinstatement following liraglutide treatment compared to vehicle	[60]
			↓ Nicotine intake and drug‐induced reinstatement following liraglutide treatment compared to vehicle	[58]
**Ghrelin pathway**				
JMV2959 (antagonist)	Mouse	In vivo microdialysis to measure dopamine in the NAc	↓ Amphetamine‐induced dopamine increase in the NAc following exenatide treatment compared to vehicle	[117]
			↓ Cocaine‐induced dopamine increase in the NAc following JVM2959 treatment compared to vehicle	[117]
			↓ Morphine‐induced dopamine increase in the NAc following JVM2959 treatment compared to vehicle	[120]
			↓ Nicotine‐induced dopamine increase in the NAc following JVM2959 treatment compared to vehicle	[114, 115]
		Drug‐induced CPP	↓ Amphetamine‐induced CPP following JVM2959 treatment compared to vehicle	[117]
			↓ Cocaine‐induced CPP following JVM2959 treatment compared to vehicle	[117]
			↓ Morphine‐induced CPP following JVM2959 treatment compared to vehicle	[120]
			↓ Nicotine‐induced CPP following JVM2959 treatment compared to vehicle	[115]
JMV2959 (antagonist)	Rat	Operant intravenous self‐administration of drugs	↓ Cocaine intake following JVM2959 treatment compared to vehicle	[107]
			↓ Methamphetamine intake following JVM2959 treatment compared to vehicle	[110]
			↓ Fentanyl intake following JVM2959 treatment compared to vehicle	[111]
			↓ Oxycodone intake following JVM2959 treatment compared to vehicle	[112]
			↓ CB receptor agonist intake following JVM2959 treatment compared to vehicle	[113]
		In vivo microdialysis to measure dopamine in the NAc	↓ Amphetamine‐induced dopamine increase in the NAc following JVM2959 treatment compared to vehicle	[87]
			↓ Fentanyl‐induced dopamine increase in the NAc following JVM2959 treatment compared to vehicle	[111]
		Drug‐induced CPP	↓ Methamphetamine‐induced CPP following JVM2959 treatment compared to vehicle	[110]
			↓ Fentanyl‐induced CPP following JVM2959 treatment compared to vehicle	[111]
			↓ THC‐induced CPP following JVM2959 treatment compared to vehicle	[113]
**Amylin pathway**				
sCT	Mouse	In vivo microdialysis to measure dopamine in the NAc	↓ Cocaine‐induced dopamine increase in the NAc following sCT treatment compared to vehicle	[153]
			↓ Nicotine‐induced dopamine increase in the NAc following sCT treatment compared to vehicle	[154]
		Drug‐induced CPP	↔ Cocaine‐induced CPP following sCT treatment compared to vehicle	[153]
			↔ Nicotine‐induced CPP following sCT treatment compared to vehicle	[154]
**HPA axis**				
Spironolactone	Mouse	Operant intravenous self‐administration	↓ Cocaine intake following spironolactone treatment compared to vehicle	[176]
Mifepristone	Mouse	Operant intravenous self‐administration	↓ Cocaine intake following mifepristone treatment compared to vehicle	[176]
	Rat	Operant self‐administration	↓ Motivation for cocaine intake (PR breaking point) following mifepristone treatment compared to vehicle	[175]
			↓ Heroin intake following mifepristone treatment compared to vehicle	[178]
			↓ Nicotine intake following mifepristone treatment compared to vehicle	[179]

*Note*: Unless otherwise stated, findings refer to outbred animal models and involve systemic, daily drug administration.

Abbreviations: CB, cannabinoid; CPP conditioned place preference; FGF‐21, fibroblast growth factor 21; FR, fixed ratio; GLP‐1, glucagon‐like peptide‐1; HPA, hypothalamic–pituitary–adrenal axis; NAc, nucleus accumbens; PR, progressive ratio; sCT, salmon calcitonin.

Human GLP‐1 studies in SUDs are limited. Blood GLP‐1 levels decreased following acute cocaine infusions in individuals with cocaine use disorder [63, 64]. However, a single dose of exenatide did not affect cocaine self‐administration or subjective effects of cocaine [64]. A small case series, in which three individuals with cocaine use disorder were treated with extended‐release exenatide, found that one of the three participants attained abstinence during the treatment period [65]. Pharmacoepidemiological evidence further suggests a lower risk of stimulant use disorder in individuals who were prescribed GLP‐1RAs [41] (Table 7). RCTs are needed to assess the efficacy of GLP‐1RAs for stimulant use disorder.

**Table 7:** Summary of key drug‐related outcomes of the human studies reviewed.

Pharmacotherapy	Study design	Outcome	References
**GLP‐1 pathway**			
Exenatide	RCT, within‐subject with nontreatment seeking individuals with cocaine use disorder (*n* = 13) received a single dose of exenatide (5 µg) before a cocaine self‐administration test, primary outcomes: number of cocaine infusions and self‐rating of euphoria and wanting	↔ Cocaine intake and subjective response following exenatide treatment compared to placebo	[64]
	An open‐label case series including three patients with cocaine use disorder who received exenatide 2 mg once weekly for 6 weeks, along with drug counseling	↓ Cocaine intake in one (reached abstinence) out of three individuals	[65]
	RCT, cigarette smoking individuals who were prediabetic and/or overweight (*n* = 83) received either exenatide 2 mg in addition to NRT once weekly during 6 weeks, primary outcome: smoking cessation, craving, and withdrawal symptoms	↓ Nicotine craving in the full sample and reduced withdrawal among abstainers following exenatide treatment compared to controls ↑ Nicotine abstinence and decreased post‐cessation weight gain following exenatide treatment compared to placebo	[70]
Liraglutide	RCT, including individuals with opioid use disorder who had recently undergone opioid withdrawal (*n* = 20) and that received either liraglutide or placebo over 3 weeks	↓ Craving for opioids in individuals receiving liraglutide compared to placebo	[66] (Preliminary results)
Dulaglutide	RCT, including people with tobacco use disorder who received either dulaglutide 1.5 mg or placebo once weekly during 12 weeks in addition to behavioral intervention and varenicline 2 mg/day, primary outcome: abstinence rate at the end of study	↔ In smoking cessation rates but decreased post‐cessation weight gain following dulaglutide treatment compared to placebo	[71]
Semaglutide	Retrospective cohort study using electronic health records from the TriNetX Analytics Network, primary outcomes: incidence and recurrence of cannabis use disorder	↓ Incidence and relapse of cannabis use disorder in individuals prescribed semaglutide compared to propensity‐score‐matched controls	[69]
	Retrospective cohort study using electronic health records from the TriNetX analytics network, primary outcome: opioid overdose in individuals with Type 2 diabetes and opioid use disorder	↓ Incidence of opioid overdose in individuals prescribed semaglutide compared to propensity‐score‐matched controls	[67]

Abbreviations: GLP‐1, glucagon‐like peptide‐1; NRT, nicotine replacement therapy; RCT, randomized controlled trial.

Clinical evidence for opioid use disorder is likewise scarce, with only one RCT that tested liraglutide in participants with opioid use disorder. The results are yet to be published, but preliminary results suggest a reduction of opioid craving [66]. A lower risk of opioid use disorder following GLP‐1RA prescription was suggested in a pharmacoepidemiological study [41]. Other pharmacoepidemiological studies have shown a lower frequency of opioid overdose in individuals who were diagnosed with opioid use disorder and prescribed GLP‐1RAs or dual agonists of the GLP‐1 and glucose‐dependent insulinotropic polypeptide (GIP) receptors [42, 67]. Preclinical evidence of the effect of GLP‐1RAs on cannabis intake is lacking, and clinical data are scarce. Lower blood GLP‐1 levels were observed after cannabis administration [68], and pharmacoepidemiological studies suggest a lower incidence and/or recurrence of cannabis use disorder in individuals who were prescribed semaglutide or other GLP‐1RAs [41, 69]. The effect of GLP‐1RAs on nicotine intake has been studied. An RCT showed more abstinence and decreased craving under exenatide as an adjunct to nicotine replacement therapy [70], but another RCT showed no effect of dulaglutide, as an adjunct to varenicline, on abstinence [71]. Both studies showed a positive effect on post‐cessation weight gain [70, 71]. Among participants who smoked, those who received semaglutide in the abovementioned alcohol RCT showed a reduction in the number of cigarettes smoked per day [45] (Table 7).

In summary, preclinical and clinical evidence of the effect of GLP‐1RAs in various SUDs is promising, but more studies are warranted to assess their tolerability, safety, efficacy, and mechanisms of action in relation to ASUDs.

## Ghrelin system

Ghrelin is an orexigenic peptide that is primarily produced by endocrine cells in the fundal part of the stomach, with smaller amounts produced by the small intestines, pancreas, and possibly brain [72]. Ghrelin has diverse physiological effects, including stimulating growth hormone release and regulating both homeostatic and hedonic feeding. The active form of ghrelin—acyl‐ghrelin (called ghrelin herein)—exerts its effect by binding to the growth hormone secretagogue receptor (GHSR)—a G protein‐coupled receptor with high intrinsic activity and expressed both peripherally and centrally, including in brain regions such as the VTA, amygdala, hippocampus, and Edinger–Westphal nucleus [73]. Peripherally produced ghrelin can influence brain pathways by acting through vagal afferents and by crossing the blood–brain barrier. Recently, liver‐expressed antimicrobial peptide 2 (LEAP2) has been identified as an endogenous GHSR inverse agonist [74] (Figure 1). The ghrelin system has been implicated in the reinforcing effects of alcohol and other addictive drugs.

### Ghrelin and alcohol use

Systemic or central ghrelin administration increased alcohol intake in rodents [75, 76]. Conversely, systemic GHSR antagonist administration decreased alcohol intake in mice, rats, and prairie voles across both acute and chronic drinking paradigms, including two‐bottle choice intermittent access, limited access, drinking‐in‐the‐dark, and operant self‐administration protocols [75, 77, 78, 79, 80, 81, 82, 83]. Furthermore, GHSR antagonism blocked relapse‐like drinking following forced abstinence in mice and rats [78, 80]. GHSR knockout mice and rats exhibited lower alcohol consumption compared with wild‐type littermates in operant self‐administration and two‐bottle choice preference tests [75, 84, 85]. Ghrelin's effects on alcohol consumption are likely mediated by central mechanisms because local GHSR antagonist administration in the LDTg and VTA decreased alcohol intake [75], whereas blocking ghrelin from crossing the blood–brain barrier via an anti‐ghrelin vaccine [82] or by pharmacologically neutralizing ghrelin [86] did not change alcohol consumption. Ghrelin signaling appears to influence alcohol consumption at least partially through interactions with the mesolimbic dopamine system. Systemic or central ghrelin administration increased extracellular dopamine levels in the NAc [76, 87, 88, 89]. This is further supported by behavioral paradigms that are used as a proxy for increases in dopamine and reward processing, where systemic or central ghrelin administration increased locomotor activity [89, 90] and induced CPP [88]. Conversely, the pharmacological or genetic blockade of ghrelin signaling (ghrelin or GHSR) attenuated alcohol‐induced dopamine release in the NAc, locomotor stimulation, and CPP [75, 76, 84, 90], further supporting a key role for ghrelin signaling in alcohol‐mediated reward (Table 4).

Central, but not systemic, administration of the endogenous GHSR inverse agonist LEAP2 decreased binge‐like alcohol consumption in mice [82] and two‐bottle choice alcohol intake in rats [91]. Central LEAP2 administration in mice also attenuated alcohol‐induced locomotor stimulation and dopamine release in the NAc [91]. These studies suggest that LEAP2 influences alcohol consumption via central mechanisms, which is consistent with the literature [82, 91] (Table 4).

Associations between ghrelin peptide and GHSR gene variants and alcohol‐related outcomes have been observed in humans [35, 92, 93, 94]. Additionally, acute alcohol intake has been shown to decrease ghrelin levels in humans [95, 96]. Following chronic alcohol consumption, human studies report higher [97, 98] or lower [99, 100] ghrelin levels; these divergent findings may be related to differences in drinking status, among other factors. Ghrelin levels have been positively correlated with alcohol craving [99, 101], and intravenous ghrelin administration increased cue‐elicited craving in individuals with AUD [102]. A follow‐up study found that intravenous ghrelin administration increased intravenous alcohol self‐administration and alcohol‐related neuronal activity in the amygdala in heavy‐drinking individuals [103]. The GHSR blocker PF‐5190457 was shown to be safe in combination with alcohol in individuals with AUD [104]. Although preliminary data in nontreatment‐seeking individuals were promising [104], a follow‐up human laboratory study did not show a significant effect of PF‐5190457 on alcohol craving, although the study was conducted in a different population of already detoxified and thus abstinent treatment‐seeking individuals with AUD. Notably, a drug effect in reducing food choice (calories) in a virtual‐reality cafeteria was observed [105] (Table 5).

To summarize, a bidirectional relationship between alcohol and the ghrelin system exists, in which alcohol influences ghrelin signaling, and ghrelin modulation, in turn, affects alcohol‐related outcomes. Preclinical evidence supports the efficacy of GHSR blockade in reducing alcohol intake, but clinical data are inconclusive, and further research is needed.

### Ghrelin and other substance use

The association between the ghrelin system and other addictive substances has been less investigated. Circulating levels of ghrelin have been shown to increase in rats following methamphetamine [106], cocaine [107], cannabis extract [108], and nicotine [109] administration. Systemic administration of the GHSR antagonist JMV2959 reduced operant intravenous self‐administration of methamphetamine [110], fentanyl [111], oxycodone [112], cocaine [107], and WIN55,212‐2, a nonselective cannabinoid receptor agonist [113] in rats. Self‐administration studies are lacking for nicotine. Ghrelin administration enhanced nicotine's ability to increase dopamine in the NAc in vitro using a superfusion method [114], whereas GHSR antagonism attenuated nicotine‐ [114, 115] and amphetamine‐induced [87] dopamine release in the NAc and VTA. Systemic or central ghrelin administration increased cocaine‐ [116] and amphetamine‐induced locomotion, whereas GHSR antagonism attenuated these effects [117]. An enhancement of cocaine‐induced CPP was observed following systemic [118] and central [119] ghrelin administration, whereas GHSR antagonism reduced cocaine‐ [117], amphetamine‐ [110, 117], morphine‐ [120], fentanyl‐ [111], tetrahydrocannabinol (THC)‐ [113], and nicotine‐induced [115] CPP. One study showed that oxycodone and cocaine self‐administration did not change blood LEAP2 levels in rodents [107, 112] (Table 6).

Genetic variations in the GHSR gene in humans have been associated with nicotine [93] and amphetamine [121] dependence. No change in serum ghrelin levels was observed following cocaine administration in people using cocaine [63] or in those with opioid use disorder [122], and no change in serum ghrelin levels was observed following cannabis administration via various routes [68]. However, an increase in total ghrelin—which measures the sum of ghrelin and des‐acyl ghrelin—was observed after oral cannabis administration [68]. The significance of this finding remains unclear; des‐acyl ghrelin does not act on GHSRs and has been shown to exert effects opposite of ghrelin, including reducing alcohol consumption in rats [123]. Another study showed that cannabis increased blood ghrelin levels in men who were immunodeficiency virus‐positive [124]. Inconsistent results have been shown following nicotine administration, with reports of both an increase [125] and no change [126] in ghrelin levels. Higher plasma levels of ghrelin were associated with smoking relapse during withdrawal [127] (Table 7).

In summary, preclinical evidence suggests that blocking GHSR may reduce the intake of various addictive substances, possibly by reducing their rewarding effects. The clinical evidence mainly focuses on genetic associations and drug‐induced changes in serum ghrelin, and reports of potential effects on craving/use are limited.

## FGF‐21 system

FGF‐21 is an endocrine hormone that is predominantly produced by the liver, with smaller amounts produced by other tissues, including the brain. Its circulating levels are primarily regulated by the liver [128]. FGF‐21 plays a crucial role in maintaining energy homeostasis, regulating metabolism, and balancing macronutrient intake [129]. Considering these functions, long‐acting FGF‐21 agonists are currently under investigation for the treatment of Type 2 diabetes and obesity. FGF‐21 also has tissue‐protective effects, particularly against metabolic and oxidative stress, and can potentially serve as a protective factor for the liver, limiting alcohol‐induced steatosis [130, 131]. FGF‐21 acts through specific receptors—primarily the FGF receptor 1c (FGFR1c), which is widely expressed in various tissues. However, the co‐receptor β‐klotho (KLB)—which is essential for FGF‐21 signaling—is primarily expressed in the liver, pancreas, and adipose tissue [132]. Centrally, KLB is expressed in the hypothalamus and brainstem, including the NTS [133], and in smaller amounts in the VTA and NAc [134]. Notably, FGF‐21 seems to cross the blood–brain barrier [135], exerting central effects that influence energy balance and macronutrient preference (Figure 1).

### FGF‐21 and alcohol use

Administration of FGF‐21 or its analogs decreased alcohol consumption in mice [128, 136] and nonhuman primates [128]. Genetic overexpression of FGF‐21 in mice had similar effects [134]. Conversely, depleting liver‐derived FGF‐21 in mice increased alcohol intake [128]. Alcohol preference also increased in mice with the brain‐specific knockout of KLB [136]. Further supporting the role of central receptors, KLB deletion in the basolateral amygdala (BLA) prevented FGF‐21 from reducing alcohol consumption in mice, and KLB‐expressing BLA neurons that project to the NAc were activated by FGF‐21 [128]. Furthermore, alcohol administration via gavage increased circulating FGF‐21 levels in mice [128, 131], an effect that was abolished in liver‐specific FGF‐21 knockout mice [128] (Table 4).

Human genetic studies support a link between FGF‐21 signaling and alcohol use, in which variations in the FGF‐21 and KLB genes have been associated with alcohol consumption [136, 137]. Consistent with preclinical findings, high circulating levels of FGF‐21 have been observed following both acute and repeated exposure to alcohol in humans [130, 131, 137, 138, 139]. Notably, FGF‐21 levels did not increase in response to alcohol cue‐reactivity in a bar‐like setting, suggesting that the elevation is induced by alcohol intake. This hypothesis was further supported by a positive correlation between FGF‐21 levels and the amount of alcohol consumption [138]. Furthermore, the alcohol‐induced increase in FGF‐21 was higher in males with AUD than in controls, suggesting a role for FGF‐21 in the pathophysiology of AUD [140].

In conclusion, the growing body of evidence that links FGF‐21 to alcohol consumption suggests that FGF‐21‐based therapies could be a promising approach for treating AUD. However, most of the extant research is preclinical, and controlled studies that assess pharmacokinetic parameters, safety, and efficacy in humans are needed. Considering the tissue‐protective effect of FGF‐21 in counteracting oxidative stress, it could benefit individuals with AUD by reducing alcohol consumption and mitigating alcohol‐induced tissue/organ damage, particularly alcohol‐induced liver injury, possibly playing a dual role in AUD and alcohol‐associated liver disease.

### FGF‐21 and other substance use

Studies that have investigated FGF‐21 in SUDs are very limited. Overexpression of FGF‐21 reduced morphine preference in mice compared with wild‐type controls [141] (Table 6). Clinically, cigarette smokers had higher levels of circulating FGF‐21 than non‐smokers [142], whereas smoking cessation did not affect FGF‐21 levels compared with those who continued to smoke [143].

## Amylin system

Amylin is a hormone primarily produced by pancreatic β cells and co‐secreted with insulin in response to nutrient intake to reduce blood glucose levels. Amylin lowers glucose levels by inhibiting glucagon secretion and slowing gastric emptying. Based on these effects, amylin receptor agonists are used to treat diabetes [144]. Amylin promotes satiety, reducing food intake and body weight, and it is thus being investigated as a target for obesity treatment. The amylin receptor is composed of the calcitonin receptor and one of three receptor activity‐modifying proteins (RAMP1‐3) [145]. Amylin receptors are expressed both peripherally and centrally in brain regions such as the NAc, VTA, LDTg, and central nucleus of the amygdala (CeA) [146] (Figure 1). Emerging evidence suggests that amylin may also modulate addictive behaviors.

### Amylin and alcohol use

In alcohol‐preferring rats chronically exposed to alcohol, both acute and repeated treatment with the amylin receptor agonist salmon calcitonin (sCT) reduced alcohol intake [147, 148]. Treatment with another amylin receptor agonist, AMY1213, decreased alcohol consumption in rats [149]. Relapse‐like drinking after forced abstinence was prevented by sCT in rats [148]. In contrast, treatment with the amylin receptor antagonist AC187 increased alcohol consumption in rats [148]. The agonist sCT also attenuated alcohol‐induced locomotor activity, dopamine release in the NAc, and CPP in mice [147]. Alcohol‐induced locomotor stimulation was reduced by sCT administration in the VTA and NAc shell, whereas alcohol‐induced dopamine release was attenuated following sCT administration in the LDTg and VTA in mice. Alcohol intake was reduced by sCT administration in the LDTg and VTA but not in the NAc shell in rats [150] (Table 4).

Preclinical data suggest that amylin agonists may reduce alcohol intake by blocking rewarding effects of alcohol. However, tolerance to both sCT and AMY1213 has been observed, which could limit their therapeutic potential [148, 149]. Published clinical studies that have examined the role of amylin in AUD are currently lacking.

### Amylin and other substance use

The role of amylin in other SUDs is less studied. Intracerebroventricular sCT administration decreased amphetamine‐induced locomotor activity in male rats [151], and intracerebroventricular amylin administration dose‐dependently reduced amphetamine‐induced locomotor activity in male rats [152]. Cocaine‐induced locomotor activity and dopamine release in the NAc were attenuated by acute systemic sCT administration in male mice, whereas cocaine‐induced CPP was unaffected [153]. Acute systemic sCT administration attenuated nicotine‐induced locomotor activity and dopamine release in the NAc shell in male mice, but no effect on CPP was detected. Repeated sCT administration prevented the acquisition of nicotine‐induced locomotor sensitization [154] (Table 6).

Cocaine administration in humans showed a trend toward reducing plasma levels of amylin in one study [63] and a significant reduction in another [64]. There are no published RCTs in this area.

## Glucocorticoids

**Fig. 2:**
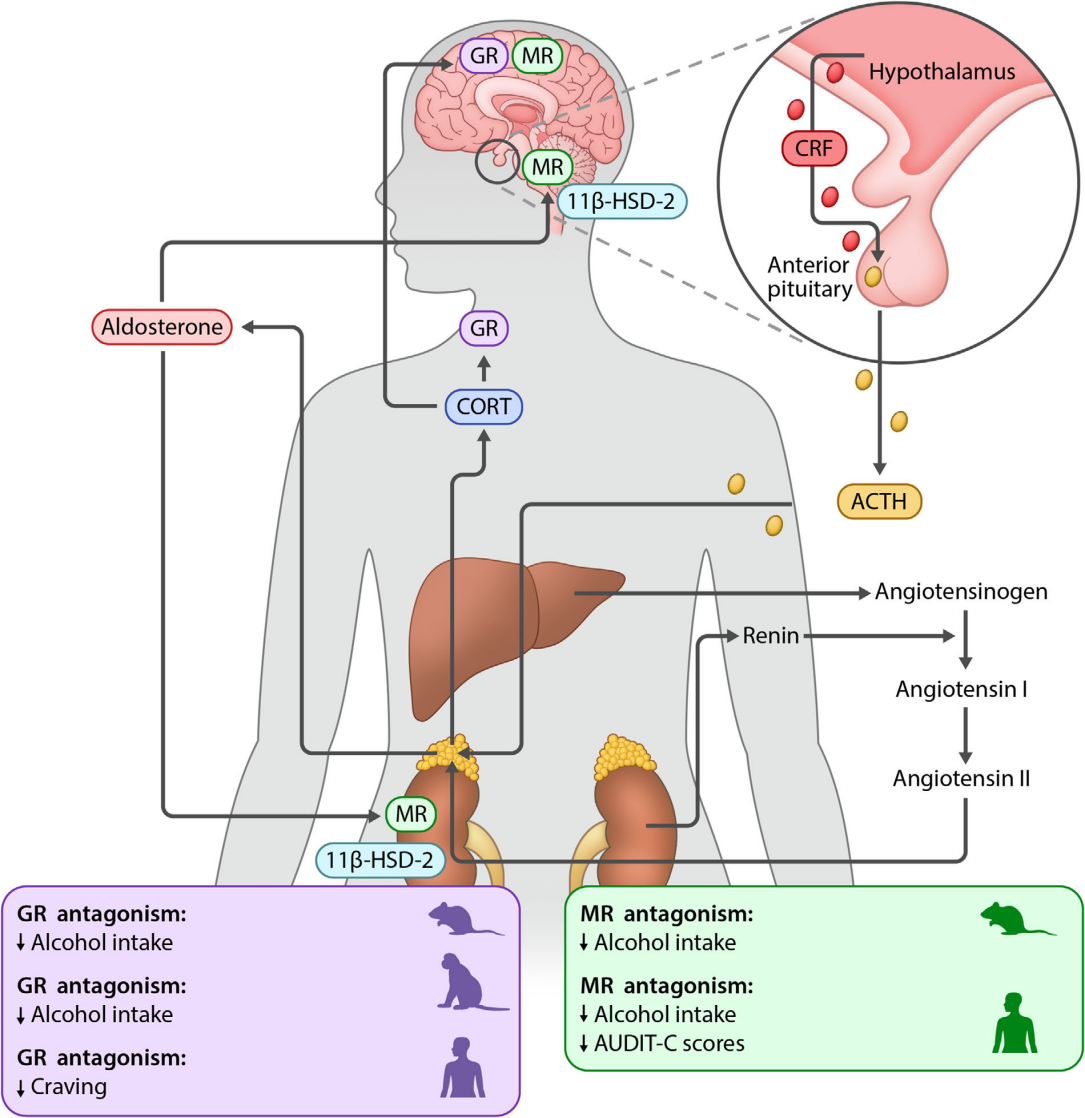
Simplified overview of the hypothalamic–pituitary–adrenal axis and its role in alcohol‐related outcomes. Both cortisol and aldosterone are produced by the adrenal glands, released into the circulation, and act via their corresponding receptors in the periphery and centrally. 11β‐HSD2, 11β‐hydroxysteroid dehydrogenase 2; ACTH, adrenocorticotropic hormone; AUDIT‐C, Alcohol Use Disorder Identification Test—Consumption; CORT, cortisol; CRF, corticotropin‐releasing factor; GR, glucocorticoid receptor; MR, mineralocorticoid receptor.

The HPA axis is the primary neuroendocrine system that regulates the body's response to stress. Activation of the HPA axis triggers the release of corticotropin‐releasing factor (CRF) from neurons in the paraventricular nucleus of the hypothalamus, which stimulates the secretion of adrenocorticotropic hormone (ACTH) from the anterior pituitary into the systemic circulation. ACTH then stimulates the adrenal gland to produce and release the steroid hormone cortisol (corticosterone in rodents). Cortisol plays a key role in adaptation to stress by promoting gluconeogenesis to support energy metabolism and inducing vasoconstriction that leads to higher blood pressure. Cortisol also has potent anti‐inflammatory properties. Its effects are mediated through both glucocorticoid receptors (GRs) and mineralocorticoid receptors (MRs). GRs are widely expressed throughout the body, including in the brain [155] (Figure 2). Stress and addiction are interconnected in a complex and bidirectional manner. Stress is a known risk factor for developing ASUDs. Alcohol and other addictive substances can acutely act as physiological stressors, activating the HPA axis [7]. Long‐term use, in turn, leads to dysregulation of the HPA axis and downstream consequences, including sensitization of extrahypothalamic stress systems [7]. Alterations of the body's stress system contribute to the pathophysiology of ASUDs and thus may be a potential treatment target. The CRF system has been extensively studied and reviewed elsewhere [156, 157]. Emerging research has focused on the involvement of GRs and MRs in ASUDs and their potential as therapeutic targets.

### Glucocorticoid receptors and alcohol use

Initial studies that indicated a role for adrenal steroid hormones in alcohol consumption showed that adrenalectomy in rats decreased alcohol intake, which was restored by administration of corticosterone but not aldosterone [158]. Systemic administration of mifepristone, a nonselective GR antagonist, dose‐dependently reduced alcohol intake in rats in a limited‐access two‐bottle choice paradigm [159]. Both mifepristone and the GR‐selective antagonist CORT113176 decreased alcohol consumption [160], and the selective GR antagonists CORT118335, CORT122928, and CORT125134 reduced operant alcohol self‐administration in rats [161]. Mifepristone and CORT113176 both decreased binge‐like alcohol intake (drinking‐in‐the‐dark paradigm) in mice that were bred to exhibit high binge‐like alcohol consumption [162]. Administration of mifepristone systemically or in the CeA decreased alcohol intake in dependent but not in nondependent rats [163] and changed GABAergic transmission in the CeA [164]. Chronic mifepristone treatment prevented alcohol self‐administration in rats and reduced alcohol self‐administration after protracted abstinence in rats with a history of dependence [165, 166]. A key role for regulatory networks downstream of the GR was identified in excessive alcohol drinking during protracted abstinence, and mifepristone administration in the NAc or VTA decreased alcohol drinking during protracted abstinence [167]. Mifepristone reduced alcohol intake in heavy‐drinking rhesus macaques [168] but not in nondependent baboons [169] (Table 4).

In humans, acute alcohol intake increases serum cortisol levels, whereas chronic alcohol consumption alters the HPA axis, resulting in higher baseline cortisol levels and blunted cortisol response following alcohol intake [170]. Three‐ to four‐fold higher cortisol concentrations were observed in hair in individuals with AUD compared with controls, suggesting chronic HPA axis hyperactivity during recent periods of heavy drinking and acute withdrawal [171]. A decrease in cue‐elicited craving was found following mifepristone treatment with [172] or without [163] pharmacologically induced stress. No changes in alcohol pharmacokinetics were observed, nor did these studies suggest safety concerns related to the administration of mifepristone in individuals with AUD [163, 172]. Mifepristone also decreased alcohol drinking in individuals with AUD and improved liver function markers that are sensitive to alcohol drinking [163] (Table 5). A novel GR antagonist, P150, also demonstrated no pharmacokinetic interactions with alcohol and was not associated with serious adverse events [173].

In summary, the nonselective GR antagonist mifepristone is effective in reducing alcohol consumption in preclinical models and alcohol craving and drinking in humans with AUD. Mifepristone appears to be safe and well tolerated in this population. The use of more selective GR antagonists may have the advantage of improving alcohol‐related outcomes while minimizing off‐target activity and side effects.

### Glucocorticoid receptors and other substance use

Consistent with the alcohol data, adrenalectomy in rats decreased cocaine self‐administration, an effect that was reversed by corticosterone administration [174]. Treatment with mifepristone reduced motivation for cocaine in a progressive‐ratio paradigm [175] and operant self‐administration of cocaine and amphetamine [176, 177] in rats. Mifepristone treatment decreased heroin intake, escalation of heroin self‐administration, and motivation to work for heroin in operant paradigms in rats and decreased self‐administration of methadone in rats and hydrocodone in zebrafish [178]. Mifepristone also decreased nicotine self‐administration in rats [179]. One study showed that mifepristone administration in the medial prefrontal cortex in male rats blocked morphine‐induced CPP [180] (Table 6).

No published RCTs are available. Transcriptional adaptations of GR signaling were found in postmortem samples of the amygdala in humans with opioid use disorder [178].

Although promising, existing evidence of GR antagonism in non‐alcohol SUDs remains limited and primarily preclinical. Additional studies are warranted to further characterize the contribution of GRs across different drug classes and to evaluate their translational potential.

## Mineralocorticoids

The renin–angiotensin–aldosterone system (RAAS) plays a central role in regulating blood volume/pressure and electrolyte balance. The RAAS is activated in response to factors such as low blood pressure and low potassium levels, leading to increased production and release of aldosterone, the primary mineralocorticoid, from the adrenal glands. Aldosterone secretion can also be stimulated by ACTH. Aldosterone exerts its effects primarily through MRs, which are expressed both peripherally and centrally (Figure 2). In the brain, MR expression is enriched in limbic regions, such as the hippocampus, amygdala, and NAc [181]. Both cortisol and aldosterone can bind to MRs. However, the enzyme 11β‐hydroxysteroid dehydrogenase 2 (11βHSD2) deactivates glucocorticoids, thereby enabling aldosterone to act on MRs in tissues where this enzyme is expressed. 11βHSD2 is highly expressed in peripheral tissues, facilitating aldosterone‐specific MR activation. In contrast, central expression of 11βHSD2 is limited, with expression primarily in the NTS [182]. Neurons in the NTS that express 11βHSD2 project to mesolimbic structures, such as the NAc. Emerging research has suggested a role for aldosterone and MRs in ASUDs [12].

### Mineralocorticoid receptors and alcohol use

Treatment with the non‐selective MR antagonist spironolactone reduced operant alcohol self‐administration in rats [183, 184] and binge‐like alcohol consumption in mice [184]. However, an early study reported no effect of spironolactone on alcohol intake in rats in a two‐bottle choice paradigm [159]. In nonhuman primates, chronic alcohol self‐administration for 6–12 months increased serum aldosterone levels [185, 186]. This was accompanied by a negative correlation between CeA expression of the MR gene and alcohol intake, suggesting brain MR downregulation in response to elevated serum aldosterone levels. Similarly, in alcohol‐dependent male rats, lower CeA MR expression correlated with compulsive‐like drinking [186]. The effect of spironolactone on alcohol consumption is thus suggested to be mediated at least partially by central MRs in the CeA. Local infusion of the MR‐specific antagonist eplerenone in the CeA and local MR gene knockdown reduced alcohol consumption in female rats [183] (Table 4).

Blood aldosterone levels were higher in individuals with active AUD compared with those who had been abstinent for 12 weeks [186], and blood aldosterone levels decreased after 12 weeks of abstinence [187]. Blood aldosterone levels appear to correlate with craving. A positive correlation was found between blood aldosterone levels and Obsessive‐Compulsive Drinking Scale scores [186, 187]. In pharmacoepidemiological studies, a decrease in weekly alcohol intake [188] and lower AUDIT‐C scores [184] were observed in those who received spironolactone for any indication compared with unexposed individuals (Table 5). Published RCTs with MR antagonists in people with AUD are lacking.

### Mineralocorticoid receptors and other substance use

Mineralocorticoid signaling may also influence the use of other addictive drugs. Spironolactone treatment decreased operant cocaine self‐administration in rats [176]. Spironolactone also reduced somatic symptoms of opioid withdrawal in both rats [189] and mice [190]. Intra‐medial prefrontal cortex spironolactone administration blocked morphine‐induced CPP in rats [180] (Table 6). Clinical studies in this area are lacking.

## Additional research venues

Alcohol and substance use can lead to neurodegeneration, potentially mediated by neuroinflammatory processes, resulting in cognitive and behavioral impairments. Immunomodulatory treatments are currently under investigation for the treatment of AUD [191]. Several candidate pharmacotherapies discussed in this review have been proposed to exert tissue‐ and/or neuroprotective effects. In addition to reducing drug intake, which may indirectly mitigate drug‐induced neurodegeneration, some treatments may yield neuroprotection through additional direct and indirect mechanisms. GLP1‐Rs are expressed not only on neurons but also on astrocytes and microglia, where their activation triggers intracellular signaling cascades associated with neuroprotection. Evidence suggests that GLP‐1RAs may slow the progression of neurodegenerative disorders and are under investigation as putative treatments for Parkinson's disease and Alzheimer's disease. FGF‐21 has tissue‐protective effects and may thus protect against alcohol‐induced hepatotoxicity. Additionally, FGF‐21 has demonstrated neuroprotective properties and may help prevent alcohol‐induced neurodegeneration. Similarly, amylin appears to have neuroprotective effects, likely through a reduction of neuroinflammation. Notably, naltrexone, an approved treatment for both AUD and opioid use disorder, is known to reduce microglial activation and exert neuroprotective effects [192]. In contrast to GLP‐1, FGF‐21, and amylin, in which agonism both reduces alcohol intake and promotes neuroprotection, ghrelin's role seems more complex. Blocking ghrelin signaling has been shown to reduce alcohol intake; however, ghrelin itself may play a neuroprotective role, with antiapoptotic properties protecting against ischemic neuronal damage and excitotoxicity.

The neuroendocrine systems reviewed herein are closely interconnected. Gut–brain peptides, such as GLP‐1 and ghrelin, modulate stress responses and energy balance. Both peptides can activate the HPA axis, leading to enhanced glucocorticoid signaling. GLP‐1‐expressing neurons project directly to CRF‐producing cells in the hypothalamus, facilitating direct activation of the HPA axis [193]. In contrast, ghrelin appears to activate CRF‐expressing neurons through indirect mechanisms that involve intermediate hypothalamic regions [194]. Stressful stimuli or administration of exogenous glucocorticoids downregulates the expression of GLP‐1 mRNA in the NTS, suggesting bidirectional regulation between GLP‐1 and the HPA axis. Conversely, stressful stimuli increase circulating ghrelin levels [195], which may further activate the HPA axis. Notably, although both GLP‐1 and ghrelin activate the HPA axis, they exert opposing effects on food and alcohol consumption. GLP‐1 suppresses food and alcohol intake, whereas ghrelin promotes these behaviors. This functional divergence highlights the complex and context‐dependent roles of gut–brain peptides, an area in need of more research.

**Fig. 3:**
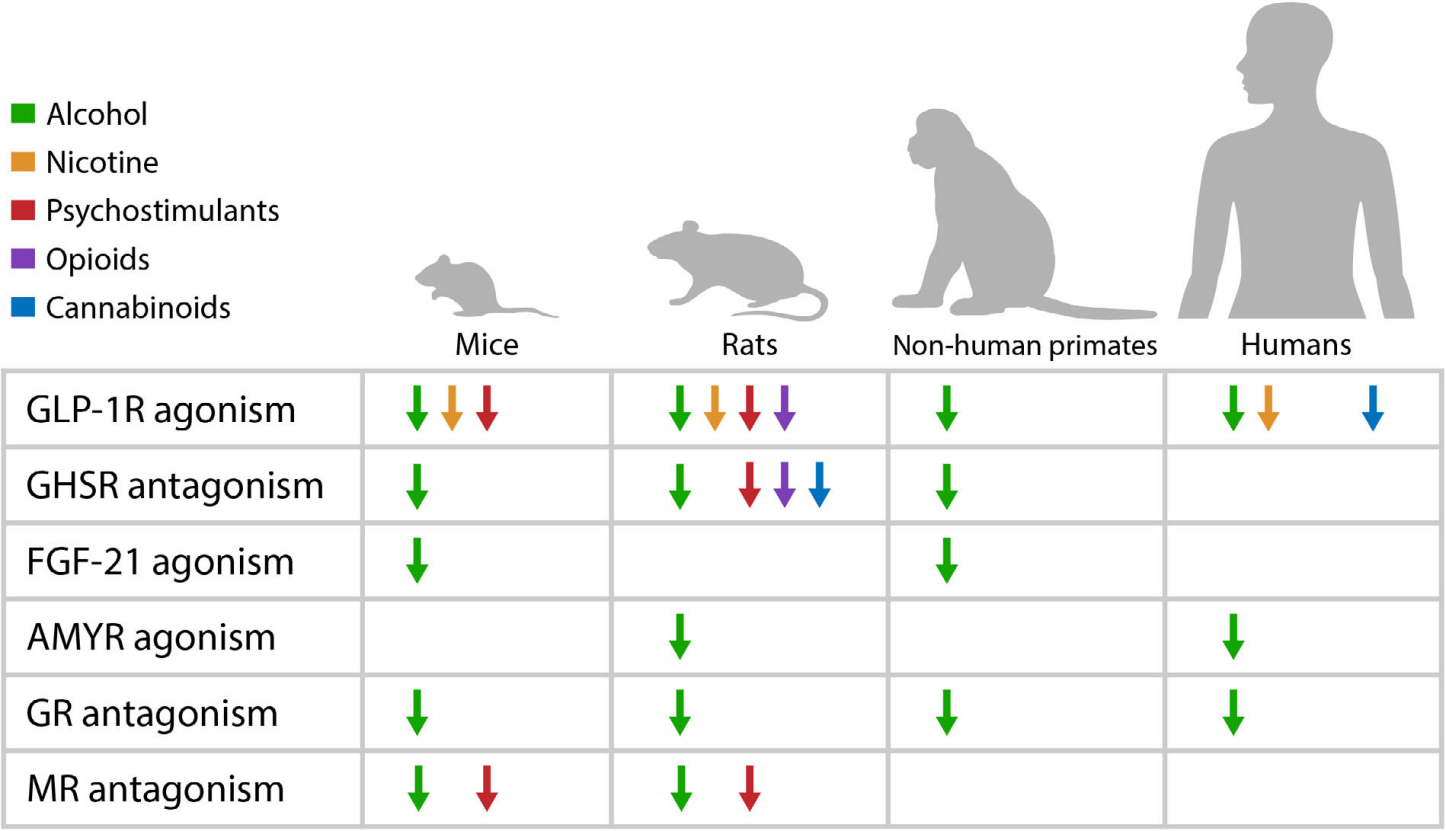
Overview of the translatability of the pharmacotherapies here reviewed in decreasing alcohol and/or other substance use. AMYR, amylin receptors; FGF‐21, fibroblast growth factor‐21; GHSR, growth hormone secretagogue receptors; GLP‐1R, glucagon‐like peptide‐1 receptor; GR, glucocorticoid receptor; MR, mineralocorticoid receptor.

The present review has limitations. We focused on putative neuroendocrine treatment targets, specifically the GLP‐1, ghrelin, FGF‐21, amylin, and HPA systems (Figures 1, 2, 3). There are other neuroendocrine systems of interest for ASUDs, such as CRF, neuropeptide Y, neurokinin‐1, and immune‐related targets, among others [156, 157, 191]. We acknowledge that as a critical narrative review, this is not meant to be a comprehensive or systematic review of all potential targets.

## Clinical implications and conclusions

An urgent need to broaden the pharmacological repertoire for the treatment of ASUDs is evident. Neuroendocrine systems could serve as novel pharmacotherapeutic targets given their involvement in the pathophysiology of these disorders. GLP‐1RAs are promising candidates for the treatment of AUD, with both preclinical and initial clinical evidence of efficacy (Figure 3). The first RCT that tested semaglutide in people with AUD showed promising results at doses lower than those usually used for diabetes and obesity [45]. Additional clinical studies—especially RCTs with semaglutide and other long‐acting GLP‐1RAs, including poly‐agonists—are needed. Tirzepatide is a long‐acting dual agonist of the GLP‐1R and GIP receptors that is approved for the treatment of diabetes, obesity, and obstructive sleep apnea. Studies of this compound and other novel GLP‐1 therapies are warranted and underway. Combining pharmacological compounds with distinct neurochemical mechanisms is an approach that has been underappreciated but is gaining more attention now. Combination therapies that aim to increase efficacy while mitigating the risk of adverse effects (e.g., by using lower doses of each medication) are appealing and should be evaluated for heterogeneous disorders, such as ASUDs, both preclinically and clinically.

The literature reviewed herein suggests that targeting neuroendocrine pathways—including gut–brain and stress pathways—could be a promising novel approach to treat ASUDs. The available literature covers alcohol and AUD to a larger extent than other substances. Alcohol is both palatable and caloric; it is possible that targeting these pathways is more favorable for alcohol than noncaloric substances. However, more studies are warranted to investigate the underlying mechanisms and assess the full potential of the reviewed pharmacotherapeutic targets.

## Conflict of interest statement

This research was supported by the Intramural Research Program of the National Institutes of Health (NIH). The contributions of the NIH authors are considered works of the United States Government. The findings and conclusions presented in this paper are those of the authors and do not necessarily reflect the views of the NIH or the US Department of Health and Human Services. Drs. Loften, Farokhnia, and Leggio are supported by NIH Intramural Research Program funding ZIA‐DA000635 (Clinical Psychoneuroendocrinology and Neuropsychopharmacology Section; PI: Dr. Lorenzo Leggio). Dr. Vendruscolo is supported by NIH Intramural Research Program funding ZIA‐DA000644 (Stress and Addiction Neuroscience Unit; PI: Dr. Leandro F. Vendruscolo), jointly supported by the National Institute on Drug Abuse, Intramural Research Program, and National Institute on Alcohol Abuse and Alcoholism, Division of Intramural Clinical and Biological Research. Dr. Loften is also supported by the Swedish Society of Medicine (SLS‐998225) and NIH Center on Compulsive Behavior.

Dr. Lorenzo Leggio, outside his federal employment at the NIH, reports honoraria from the UK Medical Council on Alcoholism (Editor‐in‐Chief of *Alcohol and Alcoholism*) and receives book royalties from Routledge. The authors have no disclosures to report.
